# Heparin-binding EGF-like growth factor enhances the activity of invasion and metastasis in thyroid cancer cells

**DOI:** 10.3892/or.2013.2659

**Published:** 2013-08-06

**Authors:** ICHIRO OTA, SHIGEKI HIGASHIYAMA, TAKASHI MASUI, KATSUNARI YANE, HIROSHI HOSOI, NARIAKI MATSUURA

**Affiliations:** 1 Department of Otolaryngology-Head and Neck Surgery, Nara Medical University, Kashihara, Nara, Japan; 2 Department of Biochemistry and Molecular Genetics, Ehime University Graduate School of Medicine, Toon, Ehime, Japan; 3 Department of Otolaryngology, Kinki University Nara Hospital, Ikoma, Nara, Japan; 4 Department of Molecular Pathology, School of Allied Health Science, Osaka University Faculty of Medicine, Suita, Osaka, Japan

**Keywords:** heparin-binding EGF-like growth factor, invasion and metastasis, thyroid cancer

## Abstract

Thyroid cancer sometimes contains poorly differentiated components, which have the potential of invasion and metastasis. We evaluated the possible roles of heparin-binding EGF-like growth factor (HB-EGF), a member of the epidermal growth factor (EGF) family, in cell growth and invasion of thyroid cancer cells, and demonstrated that HB-EGF is not only a potent mitogen but also a chemotactic factor in the thyroid cancer cells 8305C and SW579. The HB-EGF-mediated chemotaxis was inhibited by neutralizing antibody against the EGF receptor (EGFR/HER1/ErbB1) or tyrphostin AG1478, a specific inhibitor of the EGFR tyrosine kinase. The HB-EGF mRNA and protein expression was also analyzed using RT-PCR and immunofluorescence methods, respectively. In addition, in clinical immunohistochemical study, increased expression of HB-EGF and its receptors, HER1 and EGFR4 (HER4/ErbB4), was observed in thyroid carcinoma cells. Our findings suggest that HB-EGF acts as a potent paracrine and/or autocrine chemotactic factor as well as a mitogen that mediates HER1 and/or HER4 in the invasion and metastasis of thyroid carcinoma cells, including poorly differentiated papillary carcinomas or undifferentiated/anaplastic carcinomas. These data may aid in the development of novel therapeutic strategies for thyroid cancer.

## Introduction

Thyroid cancer, particularly papillary thyroid carcinoma, is one of the most common malignancies in the world and is, generally, of indolent character. Papillary thyroid carcinoma sometimes contains poorly differentiated components and has an aggressive behavior and leads to a poor prognosis (1). However, the molecular mechanism remains unclear. It was recently reported that the accumulation of multiple genetic alterations that can activate PI3K/Akt and MAPK pathways promotes thyroid carcinoma aggressiveness and progression to poorly differentiated thyroid carcinoma and undifferentiated/anaplastic thyroid carcinoma (2).

Growth factors and their specific cell surface receptors are known to play several physiological roles in cell growth and differentiation and are also involved in the development and progression of cancer through the PI3K/Akt and/or MAPK pathways (2,3). In particular, the EGF protein family, such as EGF and transforming growth factor α (TGF-α), which encompasses a number of mitogens that appear to share similar amino acid sequences, can also modulate a number of integrin-dependent functions, including cell adhesion, migration and cytoskeletal organization. However, the mechanisms underlying these phenomena are less clear.

HB-EGF is a heparin-binding member of the EGF family first identified in the condition medium of the U-937 macrophage-like cell line (4). HB-EGF is synthesized as a transmembrane precursor that can be cleaved enzymatically to release a soluble 14–20 kDa (4,5). The soluble form is a potent paracrine and/or autocrine mitogen for fibroblasts (6), smooth muscle cells (SMCs) (4,7,8), keratinocytes (9,10) and some cancer cells (11). HB-EGF has also been involved in wound healing (9) and in processes involving SMC hyperplasia such as atherosclerosis (12), pulmonary hypertension (13) and uterine leiomyomas (14). On the other hand, the transmembrane form of HB-EGF has some different functions; it works as a juxtacrine growth and adhesion factor, and uniquely it is also the receptor of diphtheria toxin (DT) (15,16).

To date, HB-EGF has been reported to be a promising therapeutic target for ovarian, breast, gastric and endometrial cancer (17,18). Although overexpression of HB-EGF is found in several types of cancer (19–21), the underlying molecular mechanisms remain unclear. Furthermore, there have been some reports on the contribution of HB-EGF in cancer metastasis and invasion of ovarian cancer cells and head and neck cancer cells (17,22). By contrast, a previous study showed that increased expression of HER4, one of the receptors of HB-EGF, was observed in thyroid papillary carcinomas compared to non-neoplastic thyroid tissues like HER1 (23). Of note, HB-EGF has been shown to be a potent chemotactic factor but not a mitogen for cell expressing HER4, in contrast to the ability of HB-EGF to stimulate both these activities in cells expressing HER1 (24).

In the present study, we evaluated the possibility of HB-EGF in cell growth and invasion of thyroid cancer cells. We demonstrated that HB-EGF was not only a potent mitogen but also a chemotactic factor in thyroid cancer cells, as previously described for fibroblasts, SMC and keratinocytes. In addition, in clinical immunohistochemical study, we also investigated the expression of HB-EGF proteins and its receptors, HER1 and HER4, in human thyroid cancer tissues, suggesting that a novel role of HB-EGF-induced chemotaxis might be mediated by tyrosine phosphorylation not only of HER1 but also of HER4 in the thyroid cancer cells.

## Materials and methods

### Reagents

HB-EGF was prepared from U-937 cell conditioned medium as previously described (4,7). EGF was obtained from R&D Systems (Minneapolis, MN, USA). Tyrphostin AG1478, a specific inhibitor of EGF receptor tyrosine kinase, was obtained from Calbiochem (La Jolla, CA, USA). RPMI-1640 was purchased from Nissui Pharmaceutical Co., Ltd., (Tokyo, Japan), fetal bovine serum (FBS) from Dainippon Pharmaceutical Co., Ltd., (Osaka, Japan) and trypsin-EDTA solution and penicillin-streptomycin solution from Gibco-BRL (Gaithersburg, MD, USA). The other chemicals and reagents are described below.

### Cells and culture

Human thyroid carcinoma cell lines, 8305C and SW579, were obtained from the Japanese Collection of Research Bioresources (JCRB) (HSRRB; Health Science Research Resources Bank, Osaka, Japan) and ATCC (Rockville, MD, USA), respectively. 8305C was derived from an undifferentiated thyroid carcinoma (25) and SW579 from a squamous cell carcinoma of thyroid (26). Cells were maintained in continuous culture at 37°C in a 5% CO_2_ humidified atmosphere. Cells were grown in RPMI-1640 medium supplemented with 10% heat-inactivated FBS. Penicillin (100 U/ml) and streptomycin (100 *μ*g/ml) were added to the media.

### Tissue samples

Thyroid tissues specimens were obtained from the patients undergoing thyroid surgery at the Nara Medical University Hospital (Kashihara, Japan). The present study was approved by the Ethics Committee of the Nara Medical University School of Medicine. Written informed consent for this study was obtained from each patient. The median age of the patients was 59.6±10.6 years (range, 29–81 years). All samples were prepared from the surgical specimens immediately after their removal from the patients and fixed in 10% buffered formalin. The histology was examined by one of the authors (M.N.), and the samples were classified according to the WHO criteria (27). For immunohistochemistry, 33 samples from 24 patients were obtained and included the following cases: 9 normal thyroids, 2 hyperplasias, 2 adenomatous goiters, 4 follicular adenomas, 3 follicular carcinomas, 11 papillary carcinomas and 2 undifferentiated carcinomas.

### Cell proliferation assays

Cell proliferation was determined by the Cell Counting Kit-8 (Dojindo, Kumamoto, Japan). 8305C cells were grown overnight in RPMI-1640 medium with 10% heat-inactivated FBS onto 96-well plates (5,000 cells/well) at 37°C in a 5% humidified atmosphere. After washing with phosphate-buffered saline (PBS), the cells were incubated in 100 *μ*l/well of serum-free RPMI-1640 medium with 0.1% bovine serum albumin (Fraction V; Sigma). 2-(2-methoxy-4-nitrophenyl)-3-(4-nitrophenyl)-5-(2,4-disulfophenyl)-2*H* tetrazolium, monosodium salt (WST-8) was added to the cells (0.5 mM/well), after 48 h of the treatment with varying concentrations of HB-EGF. The absorbance of each well was measured at 455 nm with a reference wavelength at 650 nm with MTP-32 microplate reader (Corona Electric Co., Ltd., Ibaragi, Japan). A strong correlation was confirmed between the cell proliferation by this assay and those as measured by counting the number of the cells (28).

### Migration assays

Cell migration was evaluated using a modified Boyden chamber assay (24,29,30). Eight-micron Nucleopore polyvinylpyrrolidine-free polycarbonate filters (Cambridge, MA, USA) were coated with 10.0 *μ*g/ml fibronectin (Iwaki, Chiba, Japan) in PBS for 30 min at room temperature and allowed to air dry. The filter was placed over a 48-well chamber (NeuroProbe, Cabin John, MD, USA) containing varying concentrations of HB-EGF in serum-free RPMI-1640 medium with 0.1% BSA in the lower chamber. After trypsinization, 10,000 cells in 50 *μ*l of serum-free medium were added to the wells in the upper chamber. In the checkerboard assay, varying concentrations of HB-EGF were also added to the upper chamber wells. The chamber was then placed in a 37°C with 5.0% CO_2_ humidified incubator for 3 h. Next, the upper surface of the filter was scraped to remove non-migratory cells. The filter was subsequently fixed in 10% buffered formalin for 10 min, washed with PBS and stained with hematoxylin for 10 min. Total cell number per well of the lower surface were counted visually as an index of the cell migration. Inhibition assays were conducted in a similar manner with the addition of tyrphostin AG1478 to the upper and lower chambers. The cells were incubated in serum-free medium with tyrphostin AG1478 for 1 h prior to placement in the chamber.

### Wound assay

Cell migration was also assessed by a modified *in vitro* wound assay (31). Cells were plated in complete medium (serum-free RPMI-1640 medium with 0.1% BSA) on 6-well plates. Initial plating was adjusted to yield subconfluent monolayers at the same cell density after 24 h. The monolayers were then wounded by scratching a line with a plastic scriber, and after washing with PBS, were incubated for the indicated time in the complete medium. The experiment was terminated by fixing the cells, followed by staining with hematoxylin. The distance between the advancing cells on both sides in the controls was compared with that in the presence of HB-EGF and the migratory activity was quantified by counting the cells that had migrated into the cell-free space on photographic enlargements (31–33).

### Immunohistochemistry and immunohistochemical evaluation

Immunohistochemical study for HB-EGF, HER1 and HER4 was performed using the avidin-biotin-complex (ABC) method for 9 normal thyroid tissues, 2 hyperplasias, 2 adenomatous goiters, 4 follicular adenomas, 3 follicular carcinomas, 11 papillary carcinomas and 2 undifferentiated carcinomas. Anti-HB-EGF antibody, H-1 antibody, which was generated to synthetic peptides located in cytoplasmic domains, and anti-HER4 polyclonal antibody were established by our coworker (12,19), and used at the concentration of 1:500 and 1:200, respectively. Anti-HER1 polyclonal antibody was purchased from Upstate Biotechnology, Inc., (Lake Placid, NY, USA) and applied at 1:100. Slices (4 *μ*m) of tissue section were deparaffinized and endogenous peroxidase activity was blocked with 0.3% hydrogen peroxide and 0.1% sodium azide in distilled water for 15 min. For immunohistochemistry of HER1, we performed antigen retrieval by incubating the sections with 0.03 mol/l citrate buffer (pH 6.0) and heated to 121°C for 20 min in pressure cooker. After three rinses in PBS pH 7.2 PBS, 10% bovine serum albumin (Wako, Osaka, Japan) was applied for 10 min to block the non-specific reaction. Sections were incubated with the primary antibody for 60 min at room temperature. After rinsing in PBS, they were treated with biotinylated rabbit anti-sheep IgG (Vector Laboratories, Burlingame, CA, USA) at the concentration of 1:200 for anti-HER1 antibody or biotinylated anti-rabbit IgG (Nichirei, Tokyo, Japan) at the concentration of 1:1 for anti-HB-EGF and anti-HER4 antibodies for 15 min. Again after rinsing in PBS, the sections reacted with the ABC (Dako, Copenhagen, Denmark) at the concentration of 1:300 for 15 min. The peroxidase reaction was visualized by incubating the sections with 0.02% 3,3′-diaminobenzidine tetrahydrochloride in 0.05 M Tris buffer (pH 7.6) with 0.01% hydrogen peroxide. The sections were counterstained with hematoxylin. Sections for negative control were prepared by using normal mouse serum instead of primary antibody.

We classified the results into four groups by positive cell rate: (−), 0–5% of the positive cells; (+), 5–50% of positive; (++), 50–75% of positive; (+++), 75–100% of positive.

### Immunofluorescence study

Immunofluorescence study of the transmembrane form of HB-EGF (proHB-EGF) proteins was performed with indirect immunofluorescence techniques for 8305C cells. Cells were washed with PBS and fixed with 4% paraformaldehyde. After washing in PBS, the cells were incubated with anti-HB-EGF antibody, H-1 antibody, for 30 min at room temperature. After rinsing in PBS, they were stained with fluorescein isothiocyanate washed in PBS. Cells were photographed using a fluorescence microscope (Olympus, Tokyo, Japan).

### RNA extraction

Total cellular RNA from culture cells was extracted by the acid guanidium-phenol-chloroform technique using the TRIzol (Gibco-BRL) (34). The total RNA was resuspended in diethylpyrocarbonate-treated water and stored at −80°C until use.

### RT-PCR

RT-PCR was performed as previously described (35). Single-strand cDNA was generated from 10 *μ*g of total cellular RNA in a 25-*μ*l reaction mixture containing 2.5 *μ*M oligo (dT) 18-primer, 5 mM MgCl_2_ 10 mM Tris-HCl, 50 mM KCl (pH 8.3), 1 mM d-NTP mixture, 1 U/*μ*l RNase inhibitor and 0.25 U/*μ*l AMV reverse transcriptase (Takara, Kyoto, Japan) for 1 h at 42°C. The reaction was terminated by inactivating the transcriptase at 65°C for 10 min. A 1-*μ*l aliquot of this solution was removed for subsequent first-round PCR by adding each sample to 100 *μ*l of a solution containing 2.5 mM MgCl_2_, 10 mM Tris-HCl, 50 mM KCl (pH 8.3), 200 *μ*M dATP, 200 *μ*M dCTP, 200 *μ*M dGTP, 200 *μ*M dTTP, 100 pmol of each of primer A (5′-TCCTCCAAGCCACAAGCACT-3′) and B (5′-AGAAGCCCCACGATGACCAG-3′) for HB-EGF, and 2.5 units of Taq polymerase. The initial step was 94°C for 2 min for denaturing cDNA, and then PCR was carried out for 30 cycles (30 sec at 94°C, 1 min at 55 and 1 min at 72°C). Negative controls were performed without adding the template cDNA. PCR products (10 *μ*l) were stained with ethidium bromide and analyzed by 1.5% agarose gel electrophoresis. To eliminate degraded RNA, amplification of β-actin was also performed as previously described (36).

### Diphtheria toxin (DT) sensitivity

DT sensitivity was performed for 8305C cells, since proHB-EGF is uniquely the receptor for DT (16). Approximately 10,000 cells/well were plated in a 24-well plate and incubated for 16 h. After washing each well with cold PBS, 0.5 ml/well of Ham’s F12 medium was added and the cells were exposed to DT (3.3–1,000 ng/ml) for 5 h. Subsequently, 10 *μ*l/well of ^3^H-Leu (100 *μ*Ci/ml) was added to each well and the cells were incubated for 1 h. The cells were harvested with trypsin and the extent of radiolabel incorporation was measured by beta-counter (Walla, Turku, Finland).

## Results

### Cell proliferation analysis

**Figure 1:**
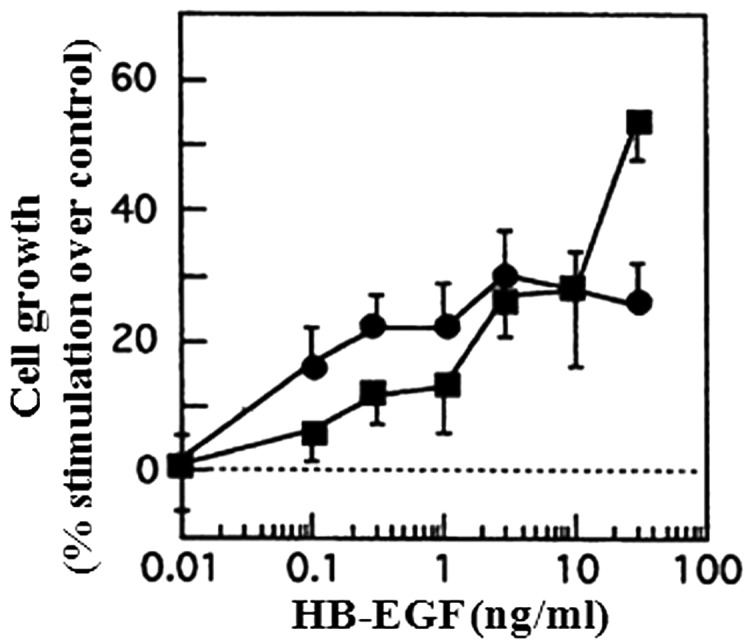
Proliferation of human thyroid cancer 8305C (closed squares) and SW579 (closed circle) cells in response to HB-EGF. These cells were grown overnight after plating in 96-well plates (5,000 cells/well) and incubated for 48 h in serum-free medium in the indicated concentrations of HB-EGF. Cell proliferation was determined by the Cell Counting Kit-8. Data are expressed as the percentage of increase compared with untreated control and represent means ± SE of triplicate determinations from three separate experiments.

EGF and TGF-α have been demonstrated to be potent mitogenic factors for thyroid carcinoma cells (23,37–39). To determine whether the cell growth of thyroid cancer 8305C and SW579 cells is modulated by HB-EGF, we studied the effects of HB-EGF on cell proliferation. HB-EGF enhanced the growth of both cell lines in a dose-dependent manner (Fig. 1). In 8305C and SW579 cells, half-maximal growth stimulation occurred at 15 and 1 ng/ml, respectively.

### Cell migration by HB-EGF

It has previously been reported that HB-EGF is a potent chemotactic factor as well as a powerful mitogenic factor for SMC (12). To test whether the cell migration of 8305C and SW579 cells is modulated by HB-EGF, a wound assay and a modified Boyden chamber assay were performed.

**Figure 2:**
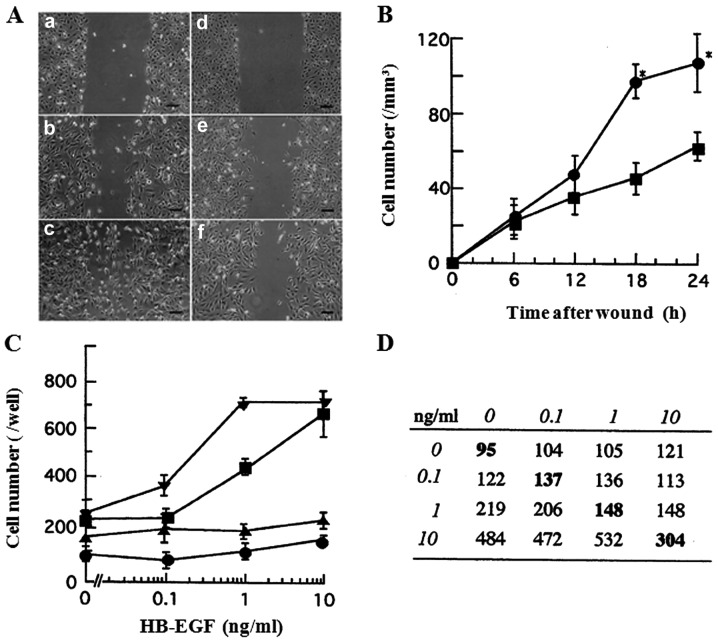
HB-EGF induces the migration activity in thyroid cancer cells. (A) In a wound assay, subconfluent monolayers of 8305C cells on 10 *μ*g/ml of fibronectin-coated plates were wounded at time 0 (a and d). The cells were allowed to migrate in the cell-free area for 12 h (b and e) or 24 h (c and f) in the presence (a–c) or the absence (d–f) of HB-EGF (10 ng/ml). Scale bar, 200 *μ*m. (B) Quantitation of cell migration in a wound assay of 8305C cells in the presence (closed circle) or the absence (closed squares) of HB-EGF (10 ng/ml). Migration was quantified by counting the cells that had migrated in the cell-free area from randomly chosen 1-mm segment of the initial wound border on photographic enlargements for 6, 12, 18 and 24 h. Data represent means ± SE of triplicate determinations from three separate experiments. Asterisks denote a significant difference (P<0.05) from the corresponding control (the absence of HB-EGF). P-values were calculated by a two-sample t-test. (C) Stimulation and inhibition by tyrphostin AG1478 of thyroid cancer cell migration in a modified Boyden chamber assay. 8305C (closed squares and closed circle) and SW579 (black down-pointing triangles and black up-pointing triangles) cells were left untreated (closed squares and black down-pointing triangles), pretreated with tyrphostin AG1478 for 1 h (closed circle and black up-pointing triangles), respectively. Migration of these cells in response to varying concentrations of HB-EGF was measured. Each point is the average of quadruplicate values. The background migration of 8305C and SW579 cells in the absence of HB-EGF was 215±20 and 245±5 cells/well, respectively. (D) Checkerboard assay. Concentrations of HB-EGF (ng/ml) added to the upper wells (horizontal lines) or lower wells (vertical lines) of Boyden chamber apparatus are shown in italics. The number of cells which migrated in the absence of a concentration gradient (same concentration of HB-EGF in the upper and lower wells) is shown in bold. This panel demonstrates a predominantly chemotactic pattern.

In a wound assay, wounds of 1 mm width were made in subconfluent monolayers of 8305C cells on 10 *μ*g/ml fibronectin-coated plates, and the cells were allowed to migrate into the cell-free area over a 24-h period (Fig. 2A). Cell migration in this assay was quantified by counting the cells that had advanced into the wounded cell-free area from a number of randomly selected 1-mm segments of the initial edge. As shown in Fig. 2B, at 6 and 12 h after the wounding, no significant differences were observed in the cell migration between the presence and the absence of HB-EGF. However, at 18 and 24 h, 10 ng/ml HB-EGF significantly stimulated the cell migration as compared to HB-EGF-free condition (P<0.05). These results indicate that HB-EGF enhances the cell migration of 8305C cells in a time-dependent manner.

In a modified Boyden chamber assay, HB-EGF induced chemotaxis of 8305C and SW579 cells at the concentrations with half-maximal stimulation of 1 and 0.3 ng/ml, respectively (Fig. 2C). Furthermore, the checkerboard assay, in which varying concentrations of HB-EGF were placed in the upper and lower wells of Boyden chamber apparatus, was performed for 8305C cells, to verify whether HB-EGF stimulates chemotaxis or chemokinesis. Cell migration to HB-EGF was predominantly consistent with chemotactic response as seen in Fig. 2D. Furthermore, when HB-EGF was present only in the upper wells, the increase in cell migration was minimal. These results indicate that HB-EGF stimulates directional chemotaxis rather than significant stimulation of random cell motility.

### Inhibition of cell migration by tyrphostin AG1478

HB-EGF has been reported to activate HER1 tyrosine phosphorylation and then to stimulate proliferation and chemotaxis in cells expressing HER1 (21). To verify whether HB-EGF stimulates HER1-mediated chemotaxis in cancer cells, we tested the inhibitory effect of tyrphostin AG1478 in HB-EGF-induced chemotaxis. In a modified Boyden chamber chemotaxis assay, the chemotactic effects of HB-EGF for 8305C and SW579 cells were markedly inhibited by the pretreatment with 100 nM tyrphostin AG1478 for 60 min prior to this assay (Fig. 2C). Furthermore, the migration with the pretreatment of tyrphostin AG1478 was more suppressed even without HB-EGF (Fig. 2C). In a wound assay, the migration was also inhibited by tyrphostin AG1478 (data not shown). These data suggest that HB-EGF-induced chemotactic effects could be mediated by HER1.

### Expression of proHB-EGF and its receptors in thyroid carcinoma cells

**Figure 3:**
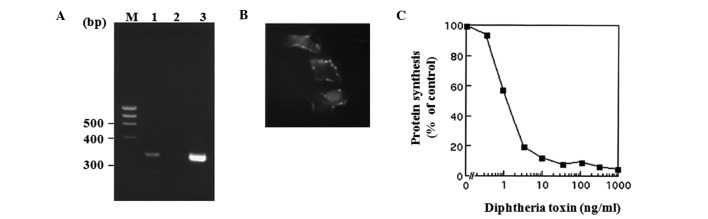
Expression of proHB-EGF in thyroid cancer cells. (A) Representative results of HB-EGF in 8305C cells (line 1), negative control (line 2) and β-actin (line 3) with RT-PCR. (B) Immunofluorescent staining of proHB-EGF in 8305C cells. (C) Diphtheria toxin (DT) sensitivity in 8305C cells. DT specifically inhibited protein synthesis in 8305C cells, indicating the presence of functioning DT receptor/proHB-EGF at the cell membrane of prostate cancer LNCaP cells.

To verify whether HB-EGF mRNA and proHB-EGF protein are expressed in 8305C cells, RT-PCR and immunofluorescence study were performed. HB-EGF mRNA expression in 8305C cells was detected with RT-PCR (Fig. 3A), and the protein expression was also observed in the cell surface by immunofluorescence staining with FITC-conjugated anti-rabbit immunoglobulin (Fig. 3B). Moreover, the functional presence of proHB-EGF protein was also confirmed by demonstration of specific sensitivity to DT (Fig. 3C), indicating that proHB-EGF can be available for DT-binding and toxin internalization as previously reported in prostate cancer cell line LNCaP cells (40). In addition, HER1 protein and mRNA expression in 8305C and SW579 cells (data not shown) were detected with both immunofluorescence study using FACSCalibur (Becton-Dickinson, San Jose, CA, USA) and RT-PCR, but HER4 was not.

### Immunohistochemistry

**Figure 4:**
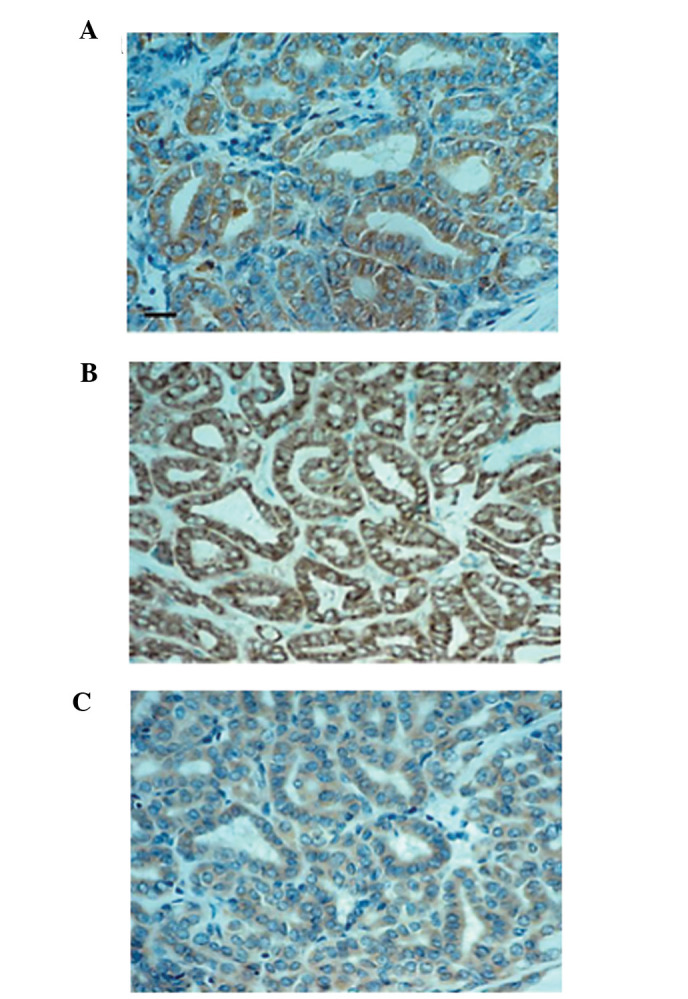
Immunohistochemical localization of HB-EGF, HER1 and HER4 in thyroid papillary carcinomas. (A) HB-EGF; (B) HER1; (C) HER4. Scale bar, 25 *μ*m.

The results of HB-EGF, HER1 and HER4 in thyroid tissues are presented in Table I. HB-EGF and HER4 staining in differentiated thyroid carcinoma tissues, such as papillary carcinomas, were localized in cytoplasm and/or cell membrane of the cells, whereas HER1 immunoreactivity was observed predominantly in cell membrane as shown Fig. 4. The intensity of HB-EGF and HER4 immunostaining in carcinomas was stronger and the number of positive cells was higher than in normal tissues. In some undifferentiated thyroid carcinoma tissues, however, HB-EGF staining was negative, although HER4 staining was strongly positive. On the other hand, HER1 was expressed widely in most malignant and benign tissues of the thyroid (Table I).

## Discussion

EGF family members, such as EGF and TGF-α, stimulate not only cell growth but also cell migration in cancer cells. Thyroid cancer cells also express EGF, TGF-α and its receptor, HER1, suggesting that they regulate thyroid cancer cell growth and invasion by autocrine and/or paracrine mechanisms (37–39,41). While HB-EGF has been shown to be a potent chemotactic factor as well as a potent mitogen for fibroblasts, SMC and keratinocytes, the effects of HB-EGF for cancer cells have been reported as follows (5,42): i) HB-EGF gene expression has been detected in a variety of tumor-derived cell lines, including prostate, breast, colon, pancreas, ovarian, head and neck carcinoma and melanoma (17,21,22); and ii) enhanced HB-EGF gene expression in tumors such as pancreatic, liver and gastric tumors, has been detected compared to normal tissues (19–21). These data suggest that it can work in an autocrine manner (21).

In the present study, we provide evidence that HB-EGF might not only be a potent mitogen but also a chemotactic factor for thyroid cancer cells, 8305C and SW579, in an autocrine and/or paracrine manner, and that the cell migration could be a chemotactic pattern as well as a chemokinetic one. These results favor the effect of HB-EGF on cancer invasion and metastasis in ovarian cancer and head and neck cancer cells (17,22). In addition, HB-EGF induced a bell shaped dose response curve in the Boyden chamber assay. These data indicate that HB-EGF could act as the soluble form (sHB-EGF) (30). The chemotactic effects of HB-EGF for 8305C and SW579 cells were markedly inhibited by tyrphostin AG1478. Cell migratory activity with the pretreatment of tyrphostin AG1478 was more suppressed than that in the absence of exogenous HB-EGF (Fig. 3), suggesting that HB-EGF could act as an autocrine chemotactic factor for thyroid carcinoma cells. ProHB-EGF protein expression was detected in 8305C and SW579 cells with immunofluorescence technique and with the demonstration of specific sensitivity to DT, and proHB-EGF mRNA expression was also detected in these cell lines. Furthermore, in immunohistochemical study, the intensity of HB-EGF immunostaining was stronger and the rate of positive cells was higher in thyroid carcinomas. Notably, the proHB-EGF immunoreactivity in undifferentiated thyroid carcinoma tissues was not always detected, suggesting that less proHB-EGF immunoreactivity in the undifferentiated carcinomas might be due to the active processing of proHB-EGF to sHB-EGF on the cell surface. In addition, undifferentiated thyroid carcinoma-derived cell line 8305C expressed HB-EGF mRNA and proHB-EGF protein (Fig. 3). These data may indicate the hypothesis as follows; when proHB-EGF can be enzymatically cleaved to rapidly release sHB-EGF in undifferentiated thyroid carcinoma cells, the tumor cells can show cell growth and migration rapidly through HER1 and/or HER4 by binding with sHB-EGF. Indeed, undifferentiated thyroid carcinomas are generally associated with poor prognosis, with most patients dying within a few months. The mechanism of its carcinogenesis, rapid growth and metastasis remains completely unclear. By contrast, differentiated thyroid carcinoma cells strongly express proHB-EGF, which might act as a tumor survival factor that induces the resistance to apoptosis due to the upregulation of p21 like hepatoma (43). Differentiated thyroid carcinomas, such as papillary carcinomas, at the earliest stage, do not always show rapid growth, while the large tumor, such as tumor size >4 cm, consists of high risk tumor factor (44), suggesting that sHB-EGF might stimulate the growth and migration of the large papillary carcinoma, after proHB-EGF could be cleaved by some proteases. It has been reported that MMP-3 cleaves HB-EGF to active sHB-EGF at a specific juxtamembrane site (45). MDC9/meltrin-g/ADAM9, a member of metalloprotease-disintegrin family, has been reported to be involved in the processing of proHB-EGF (46). Moreover, MT1-MMP co-expressed with HB-EGF in ovarian carcinoma cells has been reported to potentiate the activity of HB-EGF to promote invasive tumor growth and spreading *in vivo*(47). It is noteworthy to speculate whether some proteases including MMP-3, ADAM9 and MT1-MMP are activated in thyroid carcinoma as well as in hepatoma and ovarian carcinoma (43,47). This hypothesis in thyroid cancer favors the evidence that the production of proMMP-2 and its MT1-MMP-mediated activation in the carcinoma cell nests play an important role in the lymph node metastasis of human invasive papillary thyroid carcinomas (48–50). Furthermore, the ectodomain shedding of HB-EGF by disintegrin and metalloproteases has been reported to be a key event of receptor cross talk, as well as a novel intercellular signaling by their carboxy-terminal fragments to regulate gene expression directly (51), which could support our data that HB-EGF regulates thyroid cancer cell growth and invasion.

It has also been reported that HB-EGF can bind to HER4 as well as HER1 (24). In the present study, HER1 mRNA and protein expressions in the thyroid carcinoma cells were detected with flow cytometry and RT-PCR, but HER4 was not detected. However, in immunohistochemical study, both strong staining of HER1 and HER4 were observed in thyroid carcinoma cells including papillary carcinoma, follicular carcinoma and undifferentiated carcinoma tissues. However, HER4 tended to be more diffusely expressed in carcinomas than in benign tumor tissues, while HER1 was expressed very diffusely in most thyroid tumor tissues. This staining pattern of HER4 tended to be the same as that of HB-EGF. It has been demonstrated that papillary thyroid carcinomas express considerably higher levels of HER4 protein than non-neoplastic thyroid tissue (23), which is almost in agreement with our data. By contrast, it has been shown that HB-EGF is a chemotactic factor but not a mitogen for cells expressing HER4 in contrast to its ability to stimulate both chemotaxis and proliferation in cells expressing HER1 (24), suggesting a possible role for HER4 as well as HER1 in mediating HB-EGF-stimulated chemotaxis in thyroid carcinoma cells. By contrast, EGF and TGF-α were also found to be a potent chemotactic factors for these thyroid carcinoma cells like HB-EGF (37–39), but these growth factors are different from HB-EGF in that EGF or TGF-α binds to HER1 alone and that HB-EGF bears a heparin binding site, which binds to cell surface heparin sulfate proteoglycan, but not EGF or TGF-α. In addition, the HB-EGF-induced chemotaxis for the thyroid cells expressing HER1, but not HER4, was inhibited by tyrphostin AG1478 (Fig. 2B and C). These results suggest that HB-EGF could induce tyrosine phosphorylation of HER1 in the thyroid carcinoma cell lines expressing HER1, while in the thyroid carcinoma tissues expressing both of HER1 and HER4, HB-EGF might induce tyrosine phosphorylation of HER1 and/or HER4. Therefore, the immunohistochemical data indicate that HB-EGF-induced chemotaxis might result from tyrosine phosphorylation not only of HER1 but also of HER4 in the thyroid carcinoma cell *in vivo*. Thus, these findings suggest a novel role of HB-EGF in the invasion and metastasis of thyroid carcinoma cells.

In summary, HB-EGF is a potent chemotactic factor as well as a mitogen that mediates HER1 and/or HER4. HER4-mediated chemotaxis might induce the invasion and metastasis in thyroid carcinoma cells, particularly in poorly differentiated papillary thyroid carcinomas or undifferentiated thyroid carcinomas. Although additional studies should be carried out to establish the functional interaction between HB-EGF and HER4 as well as HER1 in thyroid carcinoma cells, these results can serve as a foundation for the development of novel therapeutic strategies including molecular targeted therapy for HB-EGF and EGFR signaling in undifferentiated thyroid carcinomas.

## Figures and Tables

**Table I:** Immunohistochemical expression of HB-EGF, HER1 and HER4 in thyroid tissues.

		HB-EGF reactivity				HER1 reactivity				HER4 reactivity			
				
Tissue diagnosis	n^a^	(−)	(+)	(++)	(+++)	(−)	(+)	(++)	(+++)	(−)	(+)	(++)	(+++)
Normal	9	5	4	0	0	0	1	1	7	6	3	0	0
Adenomatous goiter	2	0	2	3	0	0	0	0	2	1	1	0	0
Hyperplasia	2	0	0	1	1	0	0	0	2	0	1	1	0
Follicular adenoma	4	0	1	3	0	0	1	0	3	0	4	0	0
Follicular carcinoma	3	0	0	1	2	0	0	0	3	0	1	2	0
Papillary carcinoma	11	0	3	2	6	0	0	2	9	4	2	2	3
Undifferentiated/anaplastic carcinoma	2	1	0	0	1	0	0	0	2	0	0	1	1

a Number of cases. Immunoreactivity was evaluated as 0–5% (−), 5–50% (+), 50–75% (++), or 75–100% (+++) positively, according to the overall staining intensity.
